# More time for judo matches? Analysis of type of techniques, time, scores, and penalties in the Tokyo 2020 Olympic Games

**DOI:** 10.3389/fspor.2022.960365

**Published:** 2022-09-08

**Authors:** Rafael Lima Kons, Marcus F. Agostinho, João Paulo Lopes-Silva, Danilo França Conceição dos Santos, Daniele Detanico, Emerson Franchini

**Affiliations:** ^1^Department of Physical Education, Faculty of Education, Federal University of Bahia, Salvador, Bahia, Brazil; ^2^Martial Arts and Combat Sports Research Group, Sport Department, School of Physical Education and Sport, University of São Paulo, São Paulo, Brazil; ^3^Department of Physical Education, Cesmac University Center, Maceió, Brazil; ^4^Biomechanics Laboratory, Sports Centre, Federal University of Santa Catarina, Florianópolis, Santa Catarina, Brazil

**Keywords:** martial arts, competitive performance, match outcome, extra-time, technical-tactical analysis

## Abstract

This study aimed to analyze the distribution of judo matches in the Tokyo 2020 Olympic Games, considering matches in the regular duration (≤4 min) and during the extra time (the golden score) according to sex, scores, penalties, phases of competition, weight categories, and judo techniques. The data were extracted from the Official Results Books of the Olympic Games in Tokyo 2020. The combats were divided in matches that were finished in the regular duration and that were finished by the golden score, with a total of 450 matches. The Chi-square test was used to identify the association between all variables and different groups of matches duration, with the level at *p* < 0.05. The main results demonstrated significant association, considering sex and frequency of matches in the regular time (χ2 = 6.59; *p* = 0.010) for female athletes. The majority of matches ended in the 3–4 min (χ2 = 204.16; *p* < 0.001), scores were mostly awarded in the regular time and penalties in the golden score (χ2 = 48.72; *p* < 0.001), and few matches ended by the golden score for heavyweight athletes (χ2 = 15.16; *p* = 0.019). Therefore, a high number of matches ended in the golden score in the Olympic Games Tokyo 2020, with a high number of penalties in this competition.

## Introduction

In the last decade, the dynamic of official judo matches has been investigated from different perspectives (Franchini et al., 2013), involving mainly the time-motion analysis (Miarka et al., 2012, 2014), gripping actions (Calmet et al., 2010), transition from standing to the groundwork combat (Nagai et al., 2019), pacing responses during matches according to the weight category (Franchini et al., 2019), physical effort demands imposed by matches (Kons et al., 2018b), the impact of rule changes (Calmet et al., 2017a,b), the effect of video review (Kons et al., 2021), the direction of attacks (Agostinho and Franchini, 2021), the influence of penalties in the results of official matches (Balci and Ceylan, 2020), different stages on match outcomes (Ceylan et al., 2020), and the system of attack—approach, grip, displacement direction, and attack direction—in successful and unsuccessful scoring actions (Kashiwagura et al., 2021). The results of these analyses provide important indications related to the behavior of judo combats in high-level competitions, contextualizing them from different factors that influence the competitive result. Therefore, coaches can use these findings to improve the technical-tactical training organization of high-level judo athletes.

An important aspect that has influenced judo matches in the last years has been the successive rule changes over the years (Calmet et al., 2017a,b), such as the inclusion of video review (Kons et al., 2021) and changes in combat time (Calmet et al., 2017a). Additionally, an important modification that occurred was the extra time being unlimited up to the golden score or an athlete's disqualification. In the past, the golden score had the same duration as the combat time, and if the athletes did not obtain a score, the match was defined based on the referees' decision (*hantei*) [International Judo Federation (IJF), 2018—Rules]. Currently, the normal matches duration is 4 min, with varying time intervals, and the extra time is defined as overtime after the normal time (e.g., if athletes finish the match in a draw) [International Judo Federation (IJF), 2021], and, within this extra time, the golden score (currently, waza-ari or ippon), the third penalty or a direct disqualification leads to victory [International Judo Federation (IJF), 2021].

In these aspects, considering the change of time in the match duration, some matches exceed the normal combat time (currently, 4 min), often reaching two times this duration [International Judo Federation (IJF), 2021]. Considering these aspects, athletes must present good physiological and neuromuscular adaptation, especially aerobic fitness and strength endurance, to be successful in these longer matches. At the moment, studies have shown that normal-time judo matches have a high physiological demand as athletes present an elevated heart rate, blood lactate, and rating of perceived exertion values (Franchini et al., 2011, 2013) and decreased neuromuscular performance after four successive matches at normal times (e.g., < 4 min) (Detanico et al., 2015; Kons et al., 2018a). In an attempt to identify the effects of different times of judo matches on physical or technical-tactical performance in simulated judo matches, Julio et al. (2018) found higher delta blood lactate concentrations (4- and 5-min matches > 1-, 2-, and 3-min matches) and rating of perceived exertion (5-min matches > 1- and 2-min matches) in matches of longer duration compared to shorter matches; however, no differences were found for technical-tactical actions (i.e., time per sequence for standing, groundwork, combat, pause, preparation, gripping, attack, and defensive actions), demonstrating that the technical-tactical performance characteristics do not change in different match durations in simulated matches.

Considering these aspects, in official judo matches the number of matches that have been decided by the golden score has successively increased over the years [International Judo Federation (IJF), 2021; Barreto et al., 2022; Ceylan et al., 2022]. Therefore, identifying the type of techniques, scores, and penalties in high-level official matches in the golden score (*i.e*., over 4 min) can contribute to understanding the athlete's behavior in this specific condition, compared to what happens in matches finishing in the regular duration, providing valuable information for coaches concerning specific strategies to be adopted in this match phase. Thus, this study aimed to analyze the distribution of judo matches in the Tokyo 2020 Olympic Games, considering matches in the regular duration (≤ 4 min) and during the extra time (i.e., finished when a score occurred or *via* disqualification) according to sex, weight category, and phases of competitions. Additionally, we aimed to quantify match-related variables (scores, penalties, and groups of judo techniques) considering the regular duration and extra-time matches according to sex, weight category, and phases of competition. This study hypothesized that most scores would be awarded during the normal duration due to the better physical conditions at this time. Most extra-time matches would occur during the last phases of the competition when the best athletes face each other, compared to the initial phase when the seeding process allocates the top 8 higher-ranked athletes in separate seeds. Additionally, we hypothesized that no difference would be found between male and female athletes because Tokyo Olympics was the first edition to provide a similar number of places for men and women in judo competition. Finally, matches from weight categories in the middle range would have a higher prevalence of matches decided during the golden score because athletes from these categories are selected from a bigger number of athletes due to the population body mass distribution (i.e., more people in these ranges).

## Methods

### Study design

This study is a descriptive analysis of the Tokyo 2020 Olympic Games, with a focus on the matches distribution, considering the regular duration (≤4 min) and the golden score (>4 min) according to the sex, weight category, competition phases, and match-variable performance. The data for this study were extracted from the Official Results Books of the Olympic Games in Tokyo 2020 organized by the Olympic Committee of each country. The Official Results Books were documented by a judo expert referee of the International Judo Federation. Information was extracted from the Official Results Books for further analysis, such as number and type of scores, penalties, type of techniques and time analysis during the matches, and so forth. Afterward, a judo expert (a judo coach, more than 33 years of judo experience and black belt) tabulated the data, and another judo coach (more than 15 years of judo experience and black belt) performed the statistical analysis.

### Procedures

The judo combats were divided in matches that were finished in the regular duration (≤4 min; *n* = 294, 65.3%) and that were finished by the golden score (*n* = 156, 34.6%), totaling 450 matches. The total number of scores and penalties obtained during the matches was registered, considering the official referee decisions. A penalty (shido) is awarded for minor infringements, such as stepping out of the mat area or stalling the fight. Scores are awarded for successfully executed techniques. Scores are subdivided into *ippon* (the highest score, which immediately ends the match) and *waza-ari* (the medium score; two *waza-ari* scores sum up to result in *ippon*).

Judo techniques were divided into throwing techniques (*Nage-waza*) and groundwork techniques (*Katame-waza*), and then further classified according to the official technical classification [International Judo Federation (IJF), 2018]. Throwing techniques were subdivided into hand techniques (*Te-Waza*), foot techniques (*Ashi-Waza*), hip techniques (*Koshi-Waza*), and sacrifice techniques (*Mae-sutemi-waza* and *Yoko-sutemi waza*). Hand, foot, and hip techniques refer to the main body part used to throw the opponent. Sacrifice techniques are throws whereby the attacking judoka sacrifices his/her balance to execute the throw (Daigo, 2005). In addition, groundwork techniques were subdivided into immobilization techniques (*Osaekomi-Waza*), joint-locking techniques (*Kansetsu-Waza*), and choking techniques (*Shime-waza*). The phases of competition were divided into the eliminatory phases (rounds of 64–32 and rounds of 16), a quarter of finals, semi-finals, repechage, bronze contests, and finals. The weight category was divided into Extra-lightweight, Half-lightweight, Lightweight, Half-middleweight, Middleweight, Half-Heavyweight, and Heavyweight.

### Statistical analysis

The data normality was assessed by the Kolmogorov-Smirnov test. Data are presented as absolute and relative frequency, mean and standard deviation. The effect size used was Cohen's d effect size's (0.2–0.49 < small, 0.5–0.8 medium, and ≥0.8 large effects) (Cohen, 1988). Finally, independent Chi-square tests were used to test the association between different groups of match duration (regular and golden scores) and sex (male and female), weight categories, phases of competition, and match-variables performance and contingency coefficient to verify the degree of association for all variables. The significance level was set at 0.05, and all analyses were conducted using JASP software (version 0.11.1, JASP team, University of Amsterdam, Netherlands).

## Results

**Figure 1:**
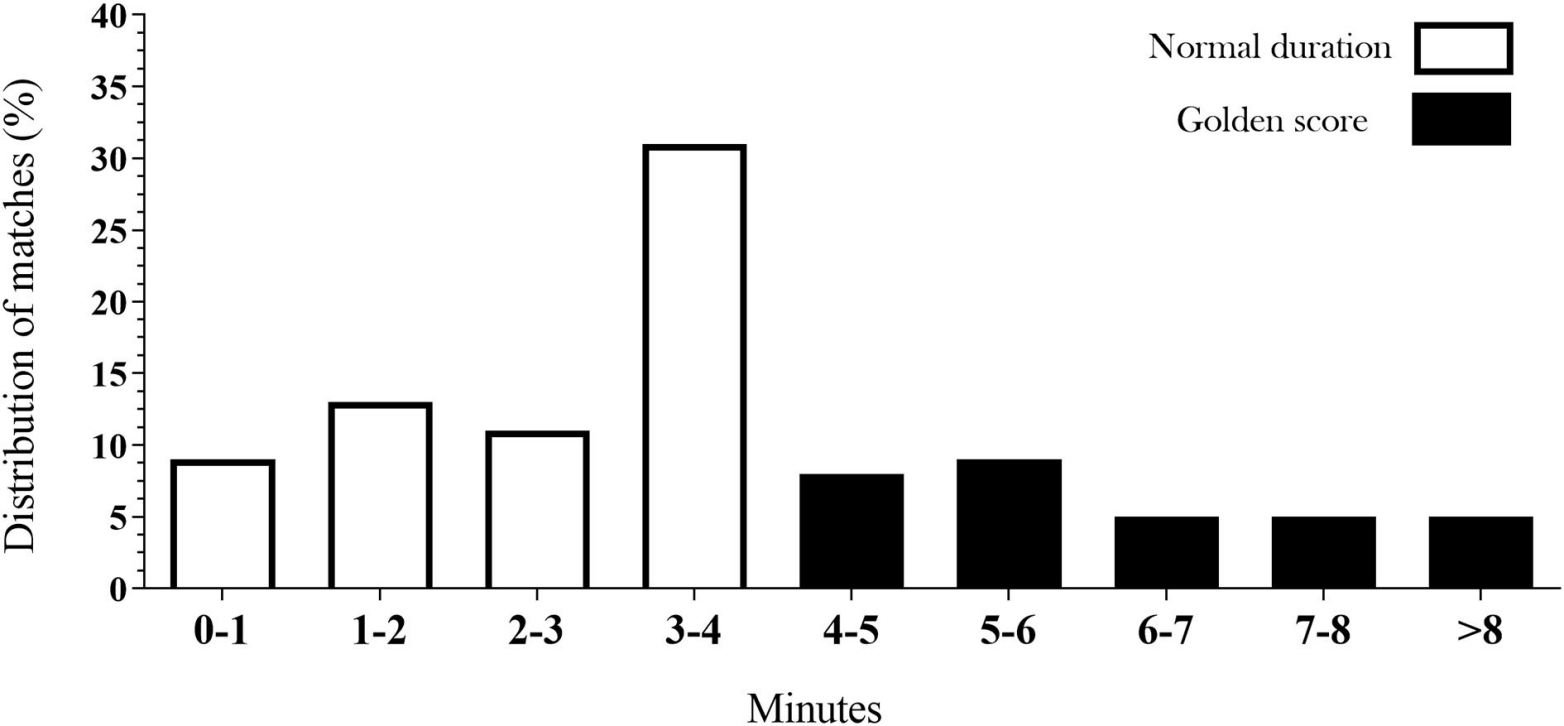
Distribution of matches according to the regular (≤4 min) and the golden score (>4 min) durations.

Significant association was detect considering sex and matches groups [$\chi_{\left(1\right)}^{2}$ = 6.59; *c* = 0.12; *p* = 0.010], with higher frequency of matches in the regular duration for women (*n* = 158, 71.1%) compared to the matches in the golden score (*n* = 64, 28.8%). For men, no difference was detected related to the matches finished in the regular duration (*n* = 136; 59.6%) and matches in the golden score (*n* = 92; 40.3%). Figure 1 shows the distributions of judo matches according to the groups of regular duration and golden score matches. Additionally, significant associations were found for the distribution of matches [$\chi_{\left(4\right)}^{2}$ = 204.16; *c* = 0.93; *p* < 0.001], with a higher percentage of matches duration between 3 and 4 min. The minimum duration in the matches occurred in the golden score was 249 s (4 min and 9 s, i.e., 9 s of extra time), and the maximum was 1,001 s (16 min and 41 s, i.e., 12 min 41 s of extra time).

Table 1 presents the matches distribution, considering different matches' duration and weight categories. A significant association was found between weight categories and groups of different matches' durations [$\chi_{\left(6\right)}^{2}$ = 15.16; *c* = 0.24; *p* = 0.019], with most matches finishing in the golden score for the half-middleweight athletes and lesser matches ending during the golden score for heavyweight athletes.

**Table 1:** Distribution of matches durations (regular and golden scores) and weight categories during the Tokyo 2020 Olympic Games.

**Weight category**	**Normal duration** ***N* and (%)**	**Golden score** ***N* and (%)**
Extra-lightweight	40 (67.7)	19 (32.2)
Half-lightweight	38 (61.2)	24 (38.7)
Light-weight	40 (57.1)	30 (42.8)
Half-middleweight	31 (40.7)	45 (59.2)
Middleweight	43 (62.3)	26 (37.6)
Half heavyweight	39 (68.4)	18 (31.5)
Heavyweight	49 (85.9)	8 (14.0)

Table 2 shows the distribution of different matches' duration and phases of competition in the Tokyo 2020 Olympic Games. No significant association was found, considering phases of competitions and different groups of matches' duration [$\chi_{\left(6\right)}^{2}$ = 10.42; *c* = 0.15; *p* = 0.166].

**Table 2:** Distribution of matches duration (regular and golden scores) and phases of competition during the Tokyo 2020 Olympic Games.

**Phases of competition**	**Normal duration** ** N and (%)**	**Golden score** ** N and (%)**
Rounds of 64-32	125 (71.4)	50 (28.5)
Round of 16	79 (68.6)	36 (31.3)
Quarterfinals	34 (55.7)	27 (44.2)
Repechage	15 (53.5)	13 (46.4)
Semi-finals	16 (55.1)	13 (44.8)
Bronze Contests	18 (64.2)	10 (35.7)
Final	7 (50.0)	7 (50.0)

Table 3 summarizes the distribution of different matches' duration and match-variables performance in the Tokyo 2020 Olympic Games. A significant association was found, considering match-variables performance and different matches' duration [$\chi_{\left(11\right)}^{2}$ = 48.72; *c* = 0.31; *p* < 0.001], demonstrating a high percentage of penalties in golden score matches.

**Table 3:** Distribution of matches considering different durations (normal duration and the golden score) and match-variables performance.

**Match-variables performance**	**Normal duration** ***N* and (%)**	**Golden score** ***N* and (%)**
*Ippon*	142 (65.4)	75 (34.5)
*Waza-ari*	177 (67.5)	85 (32.4)
Penalties	11 (26.1)	31 (73.8)
Hand techniques (*Te-waza*)	80 (68.9)	36 (31.0)
Hip techniques (*Koshi-waza*)	38 (74.5)	13 (25.0)
Foot techniques (*Ashi-waza*)	53 (55.2)	43 (44.7)
Sacrifice techniques (*Mae-sutemi-waza*)	54 (73.9)	19 (26.0)
Sacrifice techniques (*Yoko-sutemi-waza*)	8 (80.0)	2 (20.0)
Pinning techniques (*Osaekomi-waza*)	25 (83.3)	5 (16.6)
Joint locking techniques (*Kansetsu-waza*)	4 (25.0)	12 (75.0)
Choking techniques (*Shime-waza*)	6 (85.7)	1 (14.2)
*Kiken gachi* (win by forfeit)	0 (0.0)	1 (100)
*Fusen gachi* (win by opponent's abstention)	5 (83.3)	1 (16.6)

## Discussion

The hypotheses of this study were partially accepted. Specifically, the first hypothesis was confirmed as scores were mostly awarded in the regular duration. The highest frequency of matches ended in the time between 3 and 4 min, i.e., most of the matches are finished in the last minutes. The hypothesis that most extra time matches would occur during the last phases of the competition was not confirmed, as no difference was found between phases. Another hypothesis not confirmed was that regarding the absence of difference between male and female judo athletes. Conversely to our hypothesis, a higher percentage of matches was finished in the regular time compared to extra-time matches for women, whereas, for men, no difference was detected between regular and extra-time matches distribution. Finally, our last hypothesis was partially confirmed as most matches were finished with the golden score for the half-middleweight athletes, and lesser matches ended with the golden score for heavyweight athletes. Another relevant aspect to be highlighted was the high number of matches that went to the golden score in this competition (*n* = 156).

Previous investigations demonstrated, in other World judo competitions (2018 and 2019), that ~19% (Ceylan et al., 2022) and 20% (Ceylan and Balci, 2021) of matches ended in the golden score. In this study, considering the Tokyo Olympic Games, 34% of total matches ended in the golden score. Therefore, a high number of matches exceeded the normal duration time in this event, likely due to the high level of judo athletes taking part on it. Additionally, the match-variables performance demonstrated different behaviors in different groups of matches (regular duration and extra time). Specifically, most *ippon* and *waza-ari* scores occurred in the regular duration. However, a high number of penalties were found in the matches that ended in the extra time. The frequency of most judo technique groups was higher in the regular duration matches for both standing and groundwork techniques (except for joint-lock techniques). The main group of techniques used during matches was *te-waza* (hand techniques) during both regular and extra time matches. Previous studies have shown that high-level athletes perform a high frequency of hand techniques, in both recent competitions (Martins et al., 2019; Agostinho and Franchini, 2021) and, also, over previous years (Franchini and Sterkowicz, 2000). Therefore, athletes competing in the Tokyo 2020 Olympic Games also used more this type of technique. Finally, there was a small frequency of matches that ended instantly, especially *kiken gachi* in the extra time and *fusen gachi* during regular duration matches.

As hypothesized, athletes from middleweight categories would present a higher frequency of extra time matches due to the higher number of athletes in these weight categories, making the selection process likely harder for them. In our study, the half-middleweight athletes had more matches ending in the extra time, whereas heavyweight athletes presented a lower frequency of matches in the extra time. Similar to our findings, Ceylan et al. (2022) demonstrated that lighter athletes presented an increased odds ratio (1.77–2.84) of the golden score compared to the heavyweight category. Another possible explanation for these differences may also be the different pacings used by these athletes. Indeed, Franchini et al. (2019) found a higher pacing response (i.e. the ratio of low-to-high-intensity actions) for heavyweight compared to lightweight judo athletes in official competitions. This higher pace is likely to result in heavyweight matches ending before matches from lighter athletes. Additionally, heavyweight athletes presented longer duration grip disputes compared to the lighter ones (Sterkowicz-Przybycie et al., 2017). On the other hand, heavyweight athletes receive higher penalties in official competitions (Escobar-Molina et al., 2014; Calmet et al., 2017a), and this can lead to the matches finishing in the normal duration under the current rules (i.e., three penalties result in disqualification) [International Judo Federation (IJF), 2021].

Regarding the absence of differences between phases of competition, this may be related to the fact that no difference in effort phase ratio, considering technical-tactical aspects (*i.e*., approach, gripping, attack, defense, groundwork, and pause), was observed in Olympic Games matches in different phases (Miarka et al., 2018). It is important to highlight that athletes go through a rigorous selection process to participate in the Olympic Games, taking into account a competition season cycle with several world-level competitions (e.g., grand Prix, Grand Slam, and world championship) of ~4 years. From this perspective, it is expected that athletes who compete in the Olympic games present a high level of performance being similar between them, mainly related to technical-tactical (Miarka et al., 2016) and physical (Franchini et al., 2011) aspects due to the high participation of high-level competitions throughout the Olympic cycle. Therefore, matches are often decided by details, which may explain the absence of difference between phases and the high prevalence of extra-time matches during the Tokyo Olympic Games.

The high frequency of scores (*ippon* and *waza-ari*) compared to the low frequency of penalties demonstrated that athletes win most fights from the score. In this direction, Kons et al. (2018a) verified that high-level judo athletes during the Rio 2016 Olympic Games also won most of the matches by scores (especially the *ippon*, 60 to 50% for men and women, respectively), something that seems similar to the results of the present study (65.4%), considering the present Olympic Games (Tokyo 2020). Another relevant result was the high number of penalties in the matches that went into extra time. Two aspects can be speculated to explain these results. First, due to the fatigue generated by the regular match time, athletes tend to suffer penalties related to lack of combativeness or false attacks. Thus, it is likely that performance is affected by the long duration of the match before (Julio et al., 2018). On the other hand, a tactical strategy to make the opponent be penalized can also be an aspect used by athletes during extra time, since many achieve this match moment with penalties related to the previous time, and it was reported that penalties have a direct influence on the outcome of matches (Balci and Ceylan, 2020).

Finally, some limitations should be highlighted such as the lack of analysis of time motion variables determination, the number and type of transitions from standing to the groundwork combat, technical variations, and analyses, considering winners and losers. From this perspective, it is recommended that future studies consider these aspects in regular time matches and in those that went to extra time. An important aspect to be mentioned was the difficulty in adapting to the competitive environment due to the period of social isolation that affected athletes worldwide (Washif et al., 2022) and the proximity to the judo world championship with a short break (48 days) may have significantly affected the athletes' performance, causing this high number of matches that went to the extra time.

## Conclusion

Most matches in Olympic Games Tokyo 2020 were finished in the 3–4 min, a low number of matches in the heavyweight categories finished in the golden score, whereas middleweight athletes had a higher frequency of extra time matches. The competition phases did not influence the aspect related to the matches that went or not to extra time (i.e., the frequency was similar, considering the two groups). The athletes winning matches with high frequency in the normal duration used mainly *te-waza* (hand techniques), but a high number of penalties were found in matches that went to the extra time.

## Data availability statement

The raw data supporting the conclusions of this article will be made available by the authors, without undue reservation.

## Author contributions

RK, MA, DD, and EF carried out the data collection. RK and DD performed all statistical analysis. All authors conceived the study design, participated in the interpretation of data, drafted the manuscript, read, and approved the final manuscript.

## Conflict of interest

The authors declare that the research was conducted in the absence of any commercial or financial relationships that could be construed as a potential conflict of interest.

## Publisher's note

All claims expressed in this article are solely those of the authors and do not necessarily represent those of their affiliated organizations, or those of the publisher, the editors and the reviewers. Any product that may be evaluated in this article, or claim that may be made by its manufacturer, is not guaranteed or endorsed by the publisher.
